# Inhaled sevoflurane in critically ill COVID-19 patients: A retrospective cohort study

**DOI:** 10.2478/jccm-2026-0011

**Published:** 2026-01-30

**Authors:** Jose J. Zaragoza, Marco A. Baez-Garcia, Jose M. Lomeli-Teran, Daniela Anzures-Diaz, Paola Zamudio-Cantellano, Job H. Rodriguez-Guillen

**Affiliations:** Hospital H+ Queretaro, Santiago de Querétaro,Mexico; Critical Care Department, Hospital H+ Queretaro, Queretaro, Mexico; Universidad Anahuac Queretaro, Queretaro, Mexico; Colegio Mexicano de Medicina Crítica, Benito Juárez, Mexico

**Keywords:** COVID-19, sevoflurane, sedation, mechanical ventilation, intensive care unit, delirium

## Abstract

**Background:**

Managing sedation in critically ill COVID-19 patients is challenging due to high sedative requirements and organ dysfunction that alters drug metabolism. Inhaled sevoflurane offers a lung-eliminated alternative that may mitigate drug accumulation.

**Methods:**

This single-center, retrospective cohort study analyzed 43 mechanically ventilated COVID-19 patients (March–November 2020). Patients received inhaled sevoflurane adjunctive to IV sedation (n=30) or IV sedation alone (n=13). The primary outcome was the cumulative dose of IV sedatives over 7 days. Secondary outcomes included time to extubation and antipsychotic use.

**Results:**

There was no significant difference in the cumulative dose of IV sedatives between groups. However, the sevoflurane group had a significantly longer median duration of mechanical ventilation (206 [IQR 144–356] vs 144 [IQR 115–156] hours, p=0.005) and a higher requirement for antipsychotic medication (66.6% vs 15.3%, OR 18.6, p=0.011). Daily doses of propofol were lower in the sevoflurane group on specific days, but overall burden was unchanged.

**Conclusions:**

In this cohort, adjunctive inhaled sevoflurane did not significantly reduce the cumulative burden of IV sedatives and was associated with delayed extubation and increased antipsychotic use. While sevoflurane is a feasible alternative, these findings suggest caution regarding weaning and delirium management in COVID-19 patients.

## Introduction

The COVID-19 pandemic has posed an unprecedented challenge to healthcare systems worldwide. In Mexico, the impact has been particularly significant, with a high burden of hospitalizations and mortality. The burden of hospitalizations and mortality due to COVID-19 in Mexico has been significant, as detailed in several studies. From March 2020 to March 2022, Mexico experienced four epidemic waves, with 5,702,143 confirmed cases, of which 680,063 (11.9%) were hospitalized, and 324,436 (5.7%) died [1].

Managing critically ill COVID-19 patients in the intensive care unit (ICU), especially those requiring invasive mechanical ventilation, has been particularly demanding. In a tertiary care center in Mexico City, in-hospital mortality for severe COVID-19 was 30.1%, with overcrowding and lack of ICU beds contributing significantly to mortality rates [2] The need for mechanical ventilation was a critical predictor of mortality, increasing the odds of death substantially [3].

Sedation is crucial for patients undergoing mechanical ventilation to ensure tolerance of the ventilator and minimize discomfort [4,5]. However, conventional intravenous (IV) sedation is associated with several drawbacks, including hemodynamic instability, prolonged sedation, delirium, and delayed awakening [5,6]. Drug accumulation in critically ill COVID-19 patients is a complex issue, primarily driven by altered pharmacokinetics resulting from hepatic and renal impairment associated with the disease [7,8]. This accumulation leads to prolonged deep sedation, which hinders weaning from mechanical ventilation and may worsen clinical outcomes [7,8].

Inhaled anesthetics, such as sevoflurane, have emerged as an alternative approach for ICU sedation. Sevoflurane is a widely used volatile anesthetic with a rapid onset of action, easy titration, and a favorable safety profile [9]. Its primary route of elimination is through the lungs, making it particularly attractive for patients with multi-organ dysfunction [6]. Moreover, recent studies suggest that sevoflurane may have anti-inflammatory and lung-protective properties, which could be beneficial in the context of COVID-19 [10]. Other studies in the context of ARDS suggested that sevoflurane might offer benefits over benzodiazepines in terms of improved oxygenation and reduced oxidative stress in the lung, although no significant differences were observed in terms of hospital stay or mechanical ventilation-associated pulmonary damage [11,12]

This study investigated the use of inhaled sevoflurane as an adjunctive therapy for COVID-19 patients receiving invasive mechanical ventilation. The primary objective of this study was to compare the dosage of IV sedatives required for sedation, analgesia, or muscle relaxation in patients receiving inhaled sevoflurane versus those receiving conventional IV sedation. Secondary objectives included comparing the duration of mechanical ventilation, ICU length of stay, time to awakening, need for reintubation, incidence of delirium, and use of antipsychotics. We hypothesized that sevoflurane would reduce the need for IV conventional sedatives and potentially mitigate the adverse effects associated with these agents.

Given the scarcity of data on sevoflurane specifically in the COVID-19 population, this study aims to evaluate its impact not only on sedative consumption but also on critical patient-centered outcomes like extubation time and delirium surrogates.

## Methods

### Design and setting

This was a single-center, retrospective, observational cohort study conducted in the COVID-19 ICU of a tertiary care teaching hospital in Queretaro, Mexico from March 1st to November 20th, 2020. During the sanitary emergency an area for COVID patients was created where both critical and semi-critical acute patients were attended as a respiratory-ICU. The protocol was registered at clinicaltrials.gov (NCT06208592). The study was approved by the Institutional Review Board of Hospital H+ Queretaro (approval number: CEI2020a-02V1).

### Participants

All consecutive adult patients (≥18 years old) patients admitted to the respiratory ICU with confirmed SARS-CoV-2 infection during the study period were screened for eligibility. Those requiring invasive mechanical ventilation were included in the study. Exclusion criteria were patients with a known allergy to sevoflurane, those who died within 24 hours of admission, and those transferred to another hospital before extubation. In this retrospective cohort study, allocation to the sevoflurane or control group was determined by the attending physician’s clinical judgment and the availability of anesthesia conserving devices (AnaConDa) during the pandemic, rather than randomization. Sedation depth was monitored by nursing and medical staff using the Richmond Agitation-Sedation Scale (RASS), targeting a level of -2 to -4 during the acute phase of mechanical ventilation, though strict adherence was variable due to pandemic conditions.

### Data Collection and Outcomes

Baseline demographic and clinical data were collected from medical records. This included age, sex, comorbidities, laboratory values (e.g., lactate, calcium, procalcitonin), and severity of illness scores (e.g., SOFA score). The primary outcome was the cumulative dose of IV sedatives (propofol, dexmedetomidine, and opioids) administered during the first 7 days. Secondary outcomes included duration of mechanical ventilation and time to successful extubation (represented as the period since initiation of spontaneous breathing trials and successful mechanical ventilation weaning), incidence of delirium assessed using the Confusion Assessment Method for the ICU[13] (CAM-ICU) and indirectly by the prescription of antipsychotics (haloperidol, olanzapine, quetiapine, and risperidone), time to awakening (defined as), need for reintubation, and incidence of ventilator-associated pneumonia confirmed by positive sputum culture.

### Statistical analysis

The distribution of quantitative variables was examined visually using histograms and assessed for normality using the Kolmogorov-Smirnov and Shapiro-Wilk tests. Continuous variables were summarized as median and interquartile range (IQR), while categorical variables were expressed as counts and proportions or percentages. Differences in categorical variables between the sevoflurane and control groups were analyzed using Fisher’s exact test. Continuous variables were compared using the Wilcoxon rank-sum test.

An analysis for each day was performed to compare the mean daily dose of sedatives and the proportion of patients using each sedative between the two groups with Wilcoxon and Fisher’s test respectively. Linear multivariate regression analysis was performed with the cumulative dose of sedatives as the dependent variable, using sevoflurane use as the independent variable and adjusting for other potential confounders (covariates) that differed between groups at baseline with a p<0.05 in the alpha level.

Kaplan-Meier survival analysis was used to estimate the probability of successful extubation, and the log-rank test was used to compare the groups. Logistic regression analysis was performed to assess the association between sevoflurane use and binary secondary outcomes.

Variables with missing data were excluded from the analysis. Missing data were minimal and were not expected to significantly affect the results. Statistical significance was defined as P<0.05. Data was analyzed using the Stata version 16.1 (StataCorp, College Station, TX, USA).

## Results

**Fig. 1.**
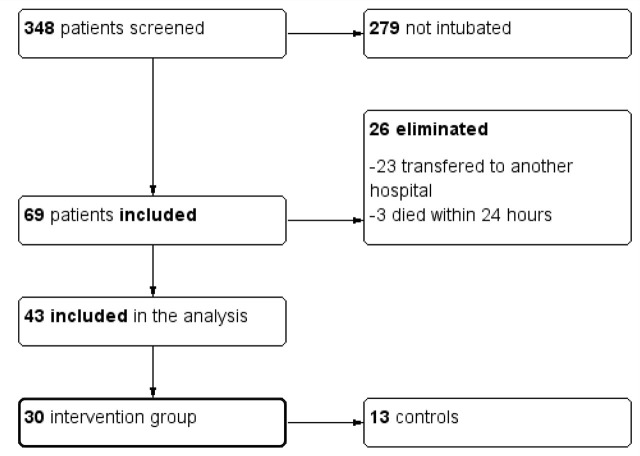
Flowchart of study population

A total of 348 patients with confirmed SARS-CoV-2 infection were admitted to the ICU during the study period. Of these, 69 required invasive mechanical ventilation. After applying the exclusion criteria, 43 patients were eligible for the study. The sevoflurane group consisted of 30 patients, while the control group included 13 patients. A flowchart of patients is presented in Figure 1.

### Demographic characteristics

Out of the 43 studied patients, 86.1% were male and 13.9% female with a median age of 53 (45–61) years. 30 patients underwent sevoflurane sedation at any time of the hospitalization and 13 underwent conventional sedation. Patients under sevoflurane sedation had a higher maximum lactate measured on day one (1.9 [1.6–2.3] *vs* 1.4 [1.3–1.6]; p=0.023), had a higher calcium (8.6 [8.3–8.9] *vs* 8.1 [7.6–8.5]; p=0.003), higher procalcitonin (0.4 [0.17–1.17] *vs* 0.105 [0.05–0.36] p=0.003) and a lower average propofol dose on day one (1.92 [1.79–2.37] *vs* 1.48 [1.05–1.93], p= 0.001. Other relevant variables such as comorbidities, severity of illness scores or crucial laboratory values were not different among groups. All patient baseline characteristics are depicted in Table 1.

**Table 1.** Baseline clinical characteristics of patients according to sevoflurane use

**Variable**	**Controls (n= 13)**	**Sevoflurane (n=30)**	**Total (n=43)**	**p value**
**Demographic characteristics**				
Age (years)	46 (40–61)	54 (48–60)	53 (45–61)	0.277
Male sex (%)	11 (84.6)	26 (86.6)	37 (86.0)	1.000
Weight (kg)	83 (75–92)	85 (75–95)	85 (75–95)	0.760
Height (m)	166 (162–175)	172 (165–177)	171 (163–177)	0.307

Systemic Hypertension (%)	4 (30.7)	8 (26.6)	12 (27.9)	1.000
Diabetes Mellitus (%)	3 (23)	10 (33.3)	13 (30.2)	0.720
Obesity (%)	7 (53.8)	16 (53.3)	23 (53.4)	1.000
COPD (%)	0	1 (3.3)	1 (2.3)	1.000
Asthma (%)	0	2 (6.6)	2 (4.6)	1.000
Sleep apnea obstructive syndrome (%)	1 (7.6)	2 (6.6)	3 (6.9)	1.000
Smoking (%)	0 (0)	1 (3.3)	1 (2.3)	1.000
Hypothyroidism (%)	2 (15.3)	0 (0)	2 (4.6)	0.086
Immunodeficiencies (%)	2 (15.3)	1 (3.3)	3 (6.9)	0.213
Hematology (%)	2 (15.3)	2 (6.6)	4 (9.3)	0.572
Chronic Heart Failure (%)	1 (7.6)	1 (3.3)	2 (4.6)	0.518
CORADS 6 (%)	2 (15.3)	10 (33.3)	12 (27.9)	0.290
Total SOFA	6 (5–7)	6 (5–7)	6 (5–7)	0.805
Respiratory SOFA	3 (2–3)	3 (3–3)	3 (3–3)	0.433
CV SOFA	3 (3–4)	3 (3–3)	3 (3–3)	0.405
Renal SOFA	0 (0–0)	0 (0–0)	0 (0–0)	0.344
Hematological SOFA	0 (0–0)	0 (0–0)	0 (0–0)	0.029*
Hepatic SOFA	0 (0–0)	0 (0–0)	0 (0–0)	0.172
Sevoflurane dose	N/A	10 (9.3–10)	10 (9.3–10)	N/A
Sevoflurane use day 1 (%)	N/A	20 (66.6%)	20 (46.5%)	0.000*
Day of symptoms at admission	9 (7–12)	9 (7–12)	9 (7–12)	0.968
Day of symptoms at intubation	12 (9–14)	9 (8–12)	10 (8–14)	0.143

**Heart Rate**				
Maximum	92 (80–100)	84 (80–93)	85 (80–96)	0.404
Minimum	62 (55–71)	57 (50–63)	58 (52–65)	0.177
Mean Arterial Pressure				
Maximum	95 (90–97)	94 (90–103)	95 (90–99)	0.915
Minimum	72 (70–74)	70 (68–73)	71 (68–74)	0.482
Maximum lactate at day one (mmol/L)	1.4 (1.3–1.6)	1.9 (1.6–2.3)	1.7 (1.4–2.3)	0.023*
PEEP maximum day 1	10 (9–12)	12 (10–12)	12 (10–12)	0.122
PaO_2_ / FiO_2_	149 (126–204)	156 (124–192)	151 (124–199)	0.801
Urinary flow (mL)	1442 (980–1890)	1652 (950–2130)	1630 (950–2130)	0.894
Hemoglobin (g/dL)	14.1 (12.8–15.4)	15.7 (14.1–16.6)	14.9 (13.9–16.5)	0.095
Leucocytes (× 10^9^/L)	7.15 (6.43–14.7)	11.9 (9.1–13.7)	11.6 (7.15–13.7)	0.272
Neutrophiles (× 10^9^/L)	5.48 (5.06–11.7)	10.2 (6.93–11.7)	9.53 (5.79–11.7)	0.095
Lymphocytes (× 10^9^/L)	.75 (.6– .94)	.99 (.74 – 1.25)	.91 (.62 – 1.23)	0.109
Platelets (× 10^9^/L)	223 (195–340)	233 (206–312)	233 (195–317)	0.926
Creatinine (mg/dL)	0.9 (0.6– 1.1)	0.7 (0.7– 0.8)	0.8 (0.7– 0.9)	0.501
BUN (mg/dL)	19 (14–24)	16 (12–21)	16 (13–23)	0.499
Sodium (mEq/L)	139 (137–141)	140 (138–142)	140 (137–142)	0.154
Potassium (mEq/L)	4.1 (3.8–4.2)	4.3 (3.9–4.7)	4.1 (3.8– 4.6)	0.217
Calcium (mg/dl)	8.1 (7.6–8.5)	8.6 (8.3–8.9)	8.5 (8.1–8.8)	0.003*
RCP (mg/L)	23.6 (19.2–36.6)	23 (16.3–35)	23.4 (16.5–36.6)	0.630
PCT (ng/mL)	0.4 (0.17–1.17)	0.105 (0.05–0.36)	0.17 (0.07–0.55)	0.003*
Total Bilirubin (mg/dL)	0.61 (0.38–.77)	0.65 (0.44–0.95)	0.65 (0.43–0.83)	0.155
Albumin (g/dL)	2.8 (2.6–3.7)	3 (2.9–3.4)	3 (2.7–3.4)	0.437
AST (U/L)	51 (41–68)	44 (32–59)	45 (33–62)	0.274
ALT (U/L)	43 (19–57)	43 (27–54)	43 (27–56)	0.663
LDH (U/L)	427 (404–512)	448 (326–513)	436 (369–513)	0.606
IL-6 (pg/mL)	69.9 (44.9–188)	104 (79–181)	102 (57–181)	0.590
D-Dimer (ng/mL)	1391 (888–1764)	850.5 (525–1883)	1099 (607–1883)	0.255
Ferritin	1423 (553.5–2815)	962.8 (649–2113)	1113 (625.3–2283)	0.737
Fibrinogen	641 (518–738)	724 (641–799)	704 (583–782)	0.064
Propofol (%)	13 (100%)	30 (100%)	43 (100%)	NT
Propofol dose on day 1 mg/kg/h	1.92 (1.79–2.37)	1.48 (1.05–1.93)	1.71 (1.09–2.12)	0.001*
Dexmedetomidine use on day 1 (%)	13 (100%)	29 (96.6%)	42 (97.6%)	1.000
Dexmedetomidine dose µg/kg/h	0.53 (0.38–0.63)	0.4 (0.34–0.6)	0.42 (0.34–0.62)	0.231
Fentanyl (%)	12 (92.3%)	26 (86.6%)	38 (88.3%)	1.000
Opioids (dose) µg/kg/min	1.21 (0.83–1.61)	0.88 (0.74 – 1.22)	0.95 (0.77–1.3)	0.078
Remifentanil use in day 1 (%)	1 (7.6%)	3 (10%)	4 (9.3%)	0.811
Remifentanil (dose)	3.37 (3.37–3.37)	1.47 (1.26–1.66)	1.56 (1.36–2.51)	0.179
Cisatracurium (dose)	-	0.1 (.09–.12)	0.1 (.09–.12)	NT
Cisatracurium use in day 1 (%)	0 (0%)	14 (46.6%)	14 (32.5%)	0.003
Rocuronium (dose)	0.44 (0.31–0.61)	0.44 (0.38–0.56)	0.44 (0.34–0.61)	0.637
Rocuronium use in day 1 (%)	6 (46.1%)	9 (30%)	15 (34.8%)	0.324
Norepinephrine (dose)	0.05 (0.02–0.13)	0.04 (0.02–0.08)	0.04 (0.02–0.10)	0.692
Norepinephrine use in day 1 (%)	12 (92.3%)	27 (90%)	39 (90.7%)	1.000
Fluid Balance on day 1 (mL)	331 (−361–350)	10.5 (−647–715)	74 (−594–700)	0.615

**Outcomes**				
ICU days	18 (16–20)	16 (13–26)	17 (13–23)	0.550
VAP (%)	7 (53.8%)	14 (46.6%)	21 (48.8%)	0.747
Delirium (%)	0 (0%)	14 (46.6%)	14 (32.5%)	0.003*
Antipsychotics (%)	2 (15.3%)	20 (66.6%)	22 (51.1%)	0.002
Mechanical Ventilation (hours)	144 (115–156)	206 (144–356)	189 (125–241)	0.005*
Mortality (%)	2 (15.3%)	4 (13.3%)	6 (13.9%)	1.000
Reintubation (%)	4 (30.7%)	4 (13.3%)	8 (18.6%)	0.217
Reintubation (hours)	46 (13.5–88.5)	42.5 (24.5–52.5)	42.5 (16–64.5)	0.772
Wake-up time (hours)	7 (5–16)	6 (1–12)	6.5 (3–13)	0.376
Propofol use (days)	6 (4–8)	7 (5–8)	7 (4–8)	0.235
Dexmedetomidine use (days)	7 (6–8)	8 (6–8)	8 (6–8)	0.314
Opioids use (days)	7 (5–8)	8 (6–8)	7 (5–8)	0.233
Neuromuscular blockage (days)	3 (0–4)	5 (3–8)	4 (2–7)	0.057

Abbreviations: COPD: Chronic Obstructive Pulmonary Disease; SOFA: Sequential Organic Failure Assessment; PEEP: Positive End Expiratory Pressure; RCP: Reactive C Protein; PCT: Procalcitonin; AST: aspartate aminotransferase; ALT: Alanine aminotransferase; LDH: Lactic dehydrogenase; IL: Interleukin.

### Primary Outcome

**Fig. 2.**
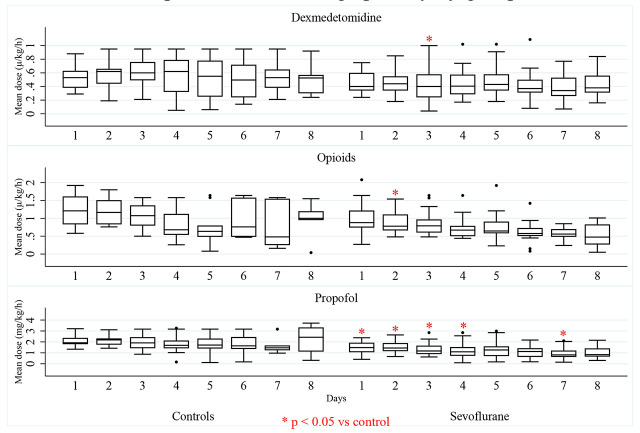
Box plot of mean daily dosage of sedatives by group in the first 7 days of follow up.

**Fig. 3.**
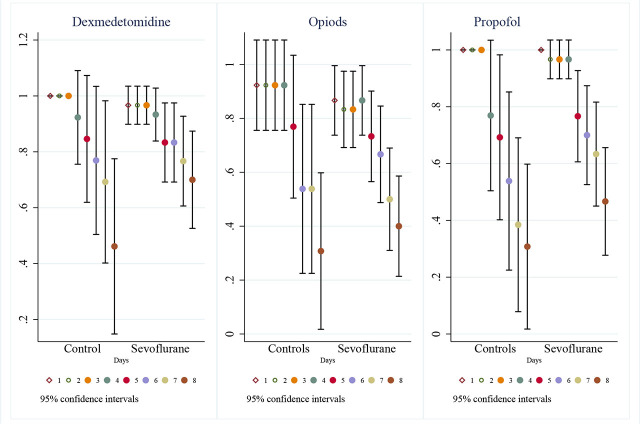
95% Confidence Interval plot of proportion of patients on every main sedative per day by group.

Detailed daily dosages are presented in Figure 2 and *Supplementary Material*. While cumulative doses over 7 days did not differ significantly, daily analysis showed statistically lower mean doses of propofol in the intervention group on days 1, 2, 3, 4, and 7 (p<0.05), and lower dexmedetomidine on day 3. In the same manner, a representation of mean proportion of patients receiving IV sedatives per day and their 95% confidence interval is represented in Figure 3. No differences were found between groups in the proportion of patients receiving each sedative every day.

**Fig. 4.**
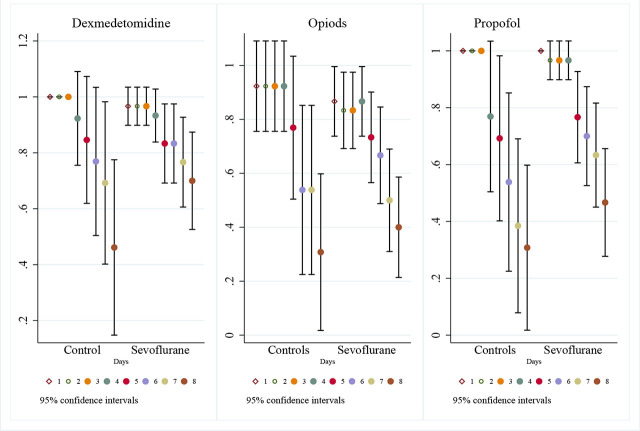
Partial-regression leverage plots or adjusted partial residual plots for primary outcome variables.

Covariates SOFA score, maximum lactate in day one, serum calcium, procalcitonin and Sevoflurane use were included in the multivariate regression model for cumulative dose of each IV sedative represented in Table 2. To consider, the use of Sevoflurane was not significantly related to either cumulative dosage or total days of use of any of the sedatives. Procalcitonin was significantly related to cumulative dose of opioids (OR 3.194 [95% CI 1.139–8.952], p=0.031). Serum calcium was correlated with cumulative dose of Propofol (OR 8.756 [95% CI 1.640–46.73], p=0.016), with total days of propofol (OR 1.729 [95% CI 1.212–2.466], p=0.003), total days of use of dexmedetomidine (OR 2.203 [95% CI 1.583–3.066], p<0.001), and total days of opioids (OR 1.768 [95% CI 1.273–2.455], p=0.001) (Table 3). An added-variable plot (a.k.a. partial-regression leverage plot or adjusted partial residual plot) using as a predictor each of the independent variables for the primary outcome is presented in Figure 4.

**Table 2.** Multiple linear regression analysis for cumulative dose for each intravenous sedative

**Opioids**	**OR**	**LCI**	**UCI**	**P**

Sevoflurane use	0.151	0.140	1.638	0.107
SOFA Score	1.088	0.459	2.579	0.829
Maximum lactate on day one	1.357	0.613	3.007	0.407
Calcium	1.968	0.898	4.309	0.082
Procalcitonin	3.194	1.139	8.952	0.031*

**Propofol**	**OR**	**LCI**	**UCI**	**P**

Sevoflurane use	0.024	0.000	15.721	0.232
SOFA Score	0.510	0.139	1.866	0.278
Maximum lactate on day one	0.861	0.024	30.82	0.929
Calcium	8.756	1.640	46.73	0.016*
Procalcitonin	14.32	0.954	214.8	0.053

**Dexmedetomidine**	**OR**	**LCI**	**UCI**	**P**

Sevoflurane use	0.813	0.173	3.808	0.783
SOFA Score	1.004	0.741	1.360	0.976
Maximum lactate on day one	1.044	0.563	3.725	0.423
Calcium	1.411	0.943	2.113	0.090
Procalcitonin	1.865	0.936	3.716	0.074

Abbreviations: SOFA: Sequential Organ Failure Assessment; OR: Odds Ratio; LCI: Lower Confidence Interval; UCI Upper Confidence Interval.

**Table 3.** Multiple linear regression analysis for total of days of use of each intravenous sedative

**Total days of Propofol use**	**OR**	**LCI**	**UCI**	**P**
Sevoflurane use	2.614	0.565	12.07	0.211
SOFA Score	1.270	0.903	1.787	0.163
Maximum lactate on day one	0.751	0.337	1.674	0.474
Calcium	1.729	1.212	2.466	0.003*
Procalcitonin	1.040	0.976	1.108	0.215
Total days of Dexmedetomidine use	OR	LCI	UCI	P
Sevoflurane use	1.246	0.299	5.181	0.756
SOFA Score	1.113	0.810	1.529	0.498
Maximum lactate on day one	0.744	0.352	1.569	0.427
Calcium	2.203	1.583	3.066	<0.001*
Procalcitonin	1.024	0.966	1.086	0.405
Total days of Opioid use	OR	LCI	UCI	P
Sevoflurane use	1.224	0.297	5.040	0.774
SOFA Score	1.219	0.889	1.672	0.209
Maximum lactate on day one	1.239	0.590	2.600	0.561
Calcium	1.768	1.273	2.455	0.001*
Procalcitonin	0.998	0.941	1.058	0.964

Abbreviations: SOFA: Sequential Organ Failure Assessment; OR: Odds Ratio; LCI: Lower Confidence Interval; UCI Upper Confidence Interval.

### Secondary outcomes

**Fig. 5.**
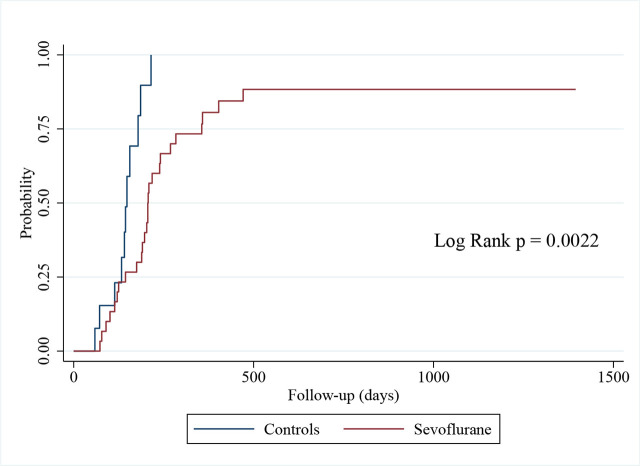
Kaplan-Meier estimates of probability of successful extubation.

Kaplan-Meier survival analysis was performed to compare the probability of successful extubation between the sevoflurane and control groups (Figure 5). The log-rank test showed a statistically significant difference (p=0.002) favoring the control group, suggesting that patients receiving conventional IV sedation had a higher probability of successful extubation.

No patients were diagnosed prospectively with delirium in the Control group, so an analysis was unfeasible. Multiple logistic regression analysis was performed to assess the association between sevoflurane use and the need for antipsychotic medication, adjusting for the same covariates as in the primary analysis, results are presented in Table 4. Sevoflurane use was independently associated with an increased risk of requiring antipsychotic medication (OR 18.682, [95% CI 1.965–177.5], p=0.011). No other covariate was independently associated with an increased risk of reintubation or ventilator-associated pneumonia. No serious adverse events related to sevoflurane administration were observed.

**Table 4.** Logistic Regression Analysis for secondary outcomes

**Antipsychotic use**	**OR**	**LCI**	**UCI**	**P**

Sevoflurane use	18.682	1.965	177.5	0.011*
SOFA Score	0.902	0.545	1.493	0.690
Maximum lactate on day one	1.073	0.422	2.724	0.882
Calcium	0.240	0.043	1.326	0.102
Procalcitonin	0.427	0.095	1.915	0.267

**Reintubacion**	**OR**	**LCI**	**UCI**	**P**

Sevoflurane use	0.679	0.095	4.813	0.699
SOFA Score	1.254	0.761	2.067	0.374
Maximum lactate on day one	1.793	0.681	4.719	0.237
Calcium	0.175	0.019	1.581	0.121
Procalcitonin	0.943	0.812	1.095	0.446

**NAVM**	**OR**	**LCI**	**UCI**	**P**

Sevoflurane use	0.912	0.164	5.049	0.917
SOFA Score	1.647	0.914	2.966	0.096
Maximum lactate on day one	1.201	0.479	3.010	0.696
Calcium	2.965	0.633	13.88	0.168
Procalcitonin	2.087	0.636	6.840	0.224

Abbreviations: SOFA: Sequential Organ Failure Assessment; OR: Odds Ratio; LCI: Lower Confidence Interval; UCI Upper Confidence Interval.

## Discussion

### Key findings

In this retrospective cohort study of critically ill COVID-19 patients, inhaled sevoflurane did not reduce the cumulative dose of IV sedatives over the first seven days compared to conventional sedation, contradicting our primary hypothesis. However, we observed transient reductions in daily propofol and dexmedetomidine requirements on specific days. Notably, sevoflurane use was associated with a longer time to successful extubation and an increased risk of requiring antipsychotic medication. Independent predictors for cumulative sedative doses included serum calcium and procalcitonin levels.

### Comparison with previous research

Our results partially align with literature suggesting volatile anesthetics can reduce opioid and sedative use during shortages[14,15]. A difference in daily dose of most IV sedatives was observed in our study, despite that the use of sevoflurane was not related to the cumulative dose of IV sedatives in the follow up time. Overall, the evidence supports the use of inhaled sevoflurane as a viable option to reduce the dose of other intravenous sedatives in mechanically ventilated COVID-19 patients, potentially improving sedation management and patient outcomes in the ICU setting.

While we observed daily dose reductions, the lack of cumulative benefit differs from randomized trials reporting faster awakening and reduced ventilation duration with sevoflurane [5,16]. These discrepancies may be due to differences in study populations, severity of illness, and sedation protocols.

Regarding delirium, existing meta-analyses suggest volatile anesthetics do not increase risk compared to IV agents [17], and may even reduce it in intracranial surgery [18] and post-cardiac arrest settings [19]. Conversely, our study found an independent association between sevoflurane and increased antipsychotic use. While direct causality is unclear, potential mechanisms involving blood-brain barrier permeability in older models have been suggested[20]. Further research specifically targeting COVID-19 cohorts is necessary to clarify this association.

We found no impact of sevoflurane on ventilator-associated pneumonia (VAP) or reintubation rates, consistent with animal models showing anti-inflammatory properties[21,22], and clinical data suggesting safety regarding respiratory parameters[23]. Interestingly, we identified a correlation between serum calcium and sedative dosage. While explored less frequently, hypocalcemia-induced neuromuscular irritability could theoretically alter sedative requirements [24]. warranting further pharmacokinetic investigation.

### Physiological Considerations in COVID-19 ARDS

A crucial factor to consider in this cohort is the severity of lung injury. Severe COVID-19 ARDS involves alveolar-capillary membrane damage, thickening, and consolidation, which can theoretically impair the uptake and elimination of volatile anesthetics. [25] In patients with severe sepsis and ARDS, the efficiency of gas exchange is compromised, potentially leading to unpredictable serum concentrations of sevoflurane compared to patients with healthy lungs, similar to other patients [26,27]. This pharmacokinetic variability might explain the prolonged time to extubation observed in our sevoflurane group, as elimination of the gas could be delayed in consolidated lung tissue, leading to a ‘wash-out’ period longer than anticipated.

### Strengths and limitations

Our study possesses several strengths. First, it provides granular, day-by-day data on sedative consumption in a real-world setting during the peak of the COVID-19 pandemic, reflecting actual clinical practice under severe resource constraints. Second, unlike studies focusing solely on drug costs or depth of sedation, we analyzed critical patient-centered outcomes, identifying crucial safety signals regarding extubation latency and delirium surrogates (antipsychotic use) that are clinically relevant for long-term ICU recovery.

However, several limitations must be acknowledged. First, the sample size was small (n=43) and unbalanced, with a limited number of control patients (n=13). This reduces the statistical power of our analysis and increases the risk of type II errors, potentially masking smaller benefits of the intervention regarding cumulative doses. Second, the single-center, retrospective design inherently introduces selection bias; allocation to the sevoflurane group was determined by device availability and clinician preference rather than randomization. Although we used multivariate regression to adjust for baseline severity confounders (e.g., SOFA score, lactate, calcium), residual confounding cannot be ruled out.

Third, while the clinical target was light-to-moderate sedation (RASS -2 to -4), the overwhelming pandemic environment precluded strict adherence to standardized sedation protocols or daily sedation interruption trials. Consequently, variations in sedation depth management between attending physicians could have influenced the time to extubation. Fourth, we lacked granular longitudinal data on the severity of sepsis or daily lung mechanics for all patients, limiting our ability to fully correlate pharmacokinetic alterations with specific degrees of alveolar-capillary damage. Finally, as an observational study, our findings establish an association but cannot prove causality between sevoflurane use and the observed delay in extubation.

## Conclusions

In this study, the use of inhaled sevoflurane as an adjunctive sedative in critically ill COVID-19 patients did not significantly reduce the cumulative dose of intravenous sedatives over the first week of ventilation, although transient reductions in daily propofol requirements were observed.

Crucially, sevoflurane use was associated with a longer time to successful extubation and an increased risk of requiring antipsychotic medication. These findings suggest that while sevoflurane is a viable alternative during sedative shortages, it requires careful monitoring of sedation depth and may present challenges during the weaning process in patients with severe respiratory failure.

Further randomized controlled trials are warranted to clarify the pharmacokinetic impact of severe lung injury on volatile anesthetics and to validate their safety profile regarding long-term neurocognitive outcomes.

## Trial registration

Clinicaltrials.gov (NCT06208592)
